# Grain Composition and Quality in Portuguese *Triticum* *aestivum* Germplasm Subjected to Heat Stress after Anthesis

**DOI:** 10.3390/plants11030365

**Published:** 2022-01-28

**Authors:** Paula Scotti-Campos, Karliana Oliveira, Isabel P. Pais, Ana Sofia Bagulho, José N. Semedo, Octávio Serra, Fernanda Simões, Fernando C. Lidon, José Coutinho, Benvindo Maçãs

**Affiliations:** 1 Instituto Nacional de Investigação Agrária e Veterinária, I.P., Quinta do Marquês, Av. República, 2784-505 Oeiras, Portugal; isabel.pais@iniav.pt (I.P.P.); jose.semedo@iniav.pt (J.N.S.); fernanda.simoes@iniav.pt (F.S.); 2 GeoBioTec, Departamento de Ciências da Terra, Faculdade de Ciências e Tecnologia, Universidade Nova de Lisboa, 2829-516 Caparica, Portugal; liana19_1@hotmail.com (K.O.); ana.bagulho@iniav.pt (A.S.B.); fjl@fct.unl.pt (F.C.L.); jose.coutinho@iniav.pt (J.C.); benvindo.macas@iniav.pt (B.M.); 3 Instituto Nacional de Investigação Agrária e Veterinária, I.P., Estrada Gil Vaz, Ap. 6, 7350-901 Elvas, Portugal; 4 Instituto Nacional de Investigação Agrária e Veterinária, I.P., Banco Português de Germoplasma Vegetal, Quinta de S. José, S. Pedro de Merelim, 4700-859 Braga, Portugal; octavio.serra@iniav.pt; 5 Earth Sciences Department, Faculdade de Ciências e Tecnologia, Universidade Nova de Lisboa, Campus da Caparica, 2829-516 Caparica, Portugal

**Keywords:** bread wheat, grain filling, high temperature, quality and nutritional traits, wholemeal flour

## Abstract

Bread wheat (*Triticum aestivum*) is a major crop worldwide, and it is highly susceptible to heat. In this work, grain production and composition were evaluated in Portuguese *T. aestivum* germplasm (landraces and commercial varieties), which was subjected to heat after anthesis (grain filling stage). Heat increased the test weight (TW) in Nabão, Grécia and Restauração, indicating an improved flour-yield potential. Mocho de Espiga Branca (MEB) and Transmontano (T94) showed higher thousand-kernel weight (TKW). Gentil Rosso presented increased soluble sugars, which are yeast substrates in the bread-making process. Ardila stood out for its protein increase under heat. Overall SDS was unaffected by higher temperature, but increased in T94, indicating a better dough elasticity for bread-making purposes. Under heat, lipid content was maintained in most genotypes, being endogenous fatty acids (FAs) key players in fresh bread quality. Lipid unsaturation, evaluated through the double bond index (DBI), also remained unaffected in most genotypes, suggesting a lower flour susceptibility to lipoperoxidation. In Grécia, heat promoted a higher abundance of monounsaturated oleic (C18:1) and polyunsaturated linoleic (C18:2) acids, which are essential fatty acids in the human diet. This work highlighted a great variability in most parameters both under control conditions or in response to heat during grain filling. Cluster analysis of traits revealed a lower susceptibility to heat during grain filling in Ardila, Restauração, and Ruivo, in contrast to MEQ, which seems to be more differentially affected at this stage. Characterization and identification of more favorable features under adverse environments may be relevant for agronomic, industrial, or breeding purposes, in view of a better crop adaptation to changing climate and an improved crop sustainability in agricultural systems more prone to heat stress.

## 1. Introduction

Bread is a staple food in many parts of the world. Eating bread helps consumers to reach their everyday needs of many nutrients. In Europe, America, and Australia, the average *per capita* consumption in 2019 stood at 30.3, 20.0, and 18.7 kg, respectively [1]. Bread not only provides energy (primarily through starch) but also contains protein, dietary fiber, and a wide array of vitamins and minerals [1]. Global warming is an unavoidable ongoing phenomenon [2] and bread wheat *(Triticum aestivum* L.) production is extremely susceptible to heat stress [3,4]. High temperature can alter biochemical, physiological, and morpho-anatomical behavior in wheat, restraining pollen viability, duration of grain filling, and starch accumulation in the endosperm [4,5]. All growth stages of wheat are sensitive to supraoptimal temperatures, with the reproductive phase being the most sensitive one as it affects both grain setting and grain filling. The most favorable temperature during flowering and grain filling ranges from 12 to 22 °C [4]. At flowering, temperature above the optimum level results in poor pollen performance and a reduced seed set [5], while postanthesis heat stress decreases starch biosynthesis and induces changes in grain composition and quality [4,6]. Modern cultivated wheat varieties have been selected for higher yields and to allow efficient agricultural practices. Landraces may depict less attractive agronomical traits, but they are a valuable source of genetic variability for breeding as regards adaptation to unfavorable environments, as well as nutritional and quality features.

In Mediterranean regions, heat waves are expected to become more frequent, fostering the search for more adapted varieties, starting from the evaluation of ancient wheats [7,8,9]. Alentejo, the major wheat producing region in Portugal, is characterized by Mediterranean climate conditions, and maximal temperatures above 32 °C are frequently observed during grain filling (March to the end of June) [6,7,8,10,11]. Photosynthesis is a key physiological process determining crop growth and yield, and very responsive to abiotic factors [12]. Changes in grain quality may occur due to modifications of photosynthetic and respiration rates, influencing the source-to-sink ratio [6,13]. The endosperm, which protects the embryo, represents 75–85% of the grain, and its major component is starch, making up 60–70% of its dry weight (DW) [14]. High temperatures inhibit starch synthase activity, hampering starch accumulation in endosperm, and decreasing grain yield [3,13,15,16]. Wheat flour contains appreciable amounts of protein (9.6%) [17]. In *T. aestivum*, bread-making quality depends on the viscoelasticity properties of dough [18]. This quality feature, evaluated through a gluten strength indicator (SDS), is determined by the amount and composition of gluten proteins in the endosperm, which can be affected by high temperatures during grain filling [4,6]. Mono- and disaccharides, which are present in whole flour in small amounts (2.3 g/100 g) [17] may vary in amount or nature, which may have implications in the use of flour for bread-making or other industrial purposes [19]. Small quantities of lipids are also found in wheat grains, usually from 2 to 6%, and play an important role in cereal processing and the nature of grain products by affecting the properties of protein and starch [1,14]. They consist mainly of oily triglycerides, phospholipids, and glycolipids, which may influence the performance of wheat flour in bread making, and the storage stability of cereals, among other features [20,21]. In grains obtained under unfavorable climate conditions, alterations in wheat lipids are likely to affect flour’s properties [21]. Quantitative and qualitative changes in lipids also depend on other factors, such as sowing date, which may interact with cultivar traits, especially in the case of total lipid and of some fatty acids (FAs) content [22]. Fas in wheat lipids are mostly polyunsaturated, which is an additional and relevant nutritional feature considering the fundamental role of essential Fas in human health [1,23,24]. However, some authors have reported a reduction in the unsaturated FAs of wheat kernels associated with selection of more productive varieties through breeding [25,26].

The aim of the present work was to explore genetic variability in some compositional grain traits and quality parameters in a set of nine Portuguese *T. aestivum* varieties grown in greenhouse under control or high-temperature conditions imposed after anthesis. Analysis of soluble sugars, lipid composition, and protein content, as well as evaluation of SDS, ash content, and color, were performed in wholegrain flour, following test weight (TW) and thousand-kernel weight (TKW) evaluation. Results will help to better characterize bread-wheat productivity and quality in response to heat, which are major concerns for farmers and breeders in the Mediterranean region.

## 2. Results

### 2.1. Test Weight (TW) and Thousand-Kernel Weight (TKW)

As regards test weight (Table 1), under control conditions Ardila and Grécia presented the highest values (77.8 and 74.1 kg/hL, respectively), while the lowest was observed in T94 (63.2 kg/hL). After heat stress, TW increased in Nabão (8%), Grécia (5%), and Restauração (4%). MEB and Ardila showed 5% and 4% decreases, respectively. The latter, despite the decrease, remained amongst genotypes presenting the highest values under stress. In the other genotypes, TWs were unaltered.

Concerning thousand-kernel weight (TKW), under control conditions Gentil Rosso presented the highest values (46.3 g), while the lowest were observed for Ruivo and T94 (*ca.* 19 g), respectively (Table 1). After heat stress, increases were observed for MEB (51.7%) and T94 (36.4%). while MEQ and Ardila showed reductions (26.2 and 12.1%, respectively). The remaining genotypes were unaltered, with Gentil Rosso still depicting the highest TKW under stress.

### 2.2. Soluble Sugars Composition

As regards the total amount of soluble sugars (Table 2), under control, MEQ depicted the highest values (4.89%), and the lowest were observed for Ardila (2.66%). This situation was maintained when comparing samples obtained under stress, and despite the strong reduction (21%) that occurred in MEQ. Nabão and Ruivo also presented decreases under heat (12% and 64%). However, heat induced an increase of total sugars (8%) in Gentil Rosso.

As regards the soluble sugars profile (Table 2), under control conditions the most abundant sugar was sucrose (1.3–2.2%), followed by raffinose (0.7–1.5%), stachyose (0.2–0.4%), glucose (0.1–0.4%), fructose (0.1–0.3%), and mannitol (0.04–0.07%). This profile was unaltered by postanthesis heat, but some changes occurred in individual sugars (Table 2). Under stress, stachyose increased in Ruivo (29%) and Ardila (18%), and decreased in all the other genotypes except in Gentil Rosso, Grécia, and Restauração, which remained unaltered. Ruivo also depicted raffinose increases (17%). Ardila, MEB, Nabão, Restauração, and T94 showed unaltered raffinose levels, unlike the remaining genotypes where decreases occurred with heat (Table 2). Sucrose increased only in Gentil Rosso (18%), and decreased in all the other varieties except Ardila, Grécia, MEB, Restauração, and T94, that presented stable values. Glucose increased in Grécia (14%) and was maintained in Ardila, Gentil Rosso, Restauração, Ruivo, and T94, decreasing in the remaining genotypes. Frutose amounts were reduced by heat in MEQ (37%), Nabão, and Ardila (22%), and were unaltered in all the other genotypes. Mannitol increased in Gentil Rosso (46%), was maintained in Ardila, Grécia, MEB, MEQ, Nabão, Ruivo, and T94, and decreased only in Restauração.

### 2.3. Protein Content, SDS Sedimentation Test, and Ash Content

Under control conditions, the highest protein percentages (*ca*. 19–20%) were observed in T94, MEQ, Grécia, and Gentil Rosso (Table 3), with the highest value being found in T94 (20.4%) and the lowest in Nabão (17.0%). As a result of heat imposition, MEB, MEQ, Nabão, and Restauração maintained stable protein values, while Gentil Rosso, Grécia, Ruivo, and T94 showed 6–14% protein decreases. Protein content increased in Ardila, presenting 6% higher values under stress.

A great variability in SDS was observed among genotypes under control conditions (22–53 mm), with the highest values (>45 mm) being obtained in Gentil Rosso, Nabão, Restauração, and MEB (Table 3). SDS values were unaltered by heat stress, except in T94, which presented an increase of 18%.

Under control conditions, ash values varied between 1.9 and 2.4% (Table 3). Heat stress did not affect the majority of the genotypes, but ash percentage decreased by 16% in Nabão and 11% in Ardila and Grécia. 

### 2.4. Lipid Profile

**Figure 1:**
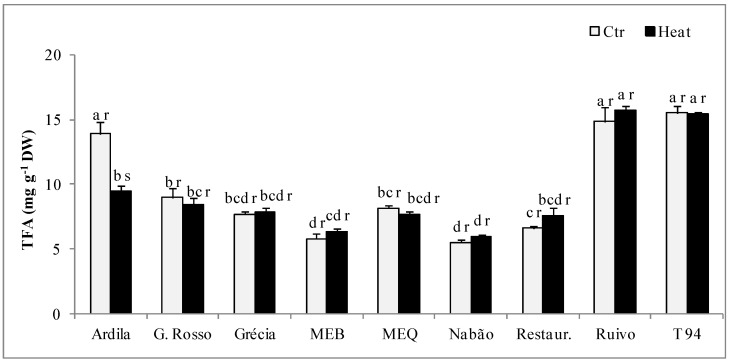
Total fatty acids (TFA) in wholemeal flour obtained from the grains of nine bread-wheat genotypes under control conditions (Ctr) or subjected to heat after anthesis. Mean values ± SE (*n* = 6). Different letters express significant differences between genotypes for each treatment (a, b, c, d) or between control and heat treatment for the same genotype (r, s); a and r represent the highest values.

As regards total fatty acids (TFA), under control conditions the highest values (*ca*. 14–15 mg g^−1^ DW) were observed for Ardila, Ruivo, and T94 (Figure 1). MEB and Nabão depicted the lowest TFA amounts (*ca*. 7 mg g^−1^ DW). Ardila was the only genotype affected by heat, showing a 32% decrease in relation to control.

Concerning individual fatty acids profile (Table 4), C18:2 (linoleic acid) was the most abundant, followed by C16:0 (palmitic acid), C18:1 (oleic acid), C18:3 (linolenic acid), C18:0 (stearic acid), and C20:1 (gadoleic acid). This pattern was unaltered by heat treatment, although some changes occurred in several FAs, according to genotypes. Kernels developed under high temperatures showed decreased percentages of C18:2 (Ardila and Gentil Rosso) and C16:0 (Grécia). Higher relative abundance was observed for C18:2 (Grécia), C18:1 (Gentil Rosso, Grécia, and T 94), and C18:0 (Gentil Rosso and T94).

As regards lipids unsaturation, expressed through a double bond index (DBI), no differences were found between genotypes under control conditions. As a result of post-anthesis heat, DBI remained unaltered in all gentoypes except Grécia, which showed a significant increase (17%) in relation to control (Table 4).

### 2.5. Color Measurements

In wholegrain samples, lightness (Table 5), L* values ranged from 52 to 57 under control conditions, with the highest being observed in Gentil Rosso, Grécia, MEQ, Restauração, and T94 (>55). After heat stress, L* increased in Gentil Rosso (7%) and Ruivo (5%), and decreased in MEQ (3%). Coordinates a* and b*, related to chromaticity, were not affected by heat, and differences between genotypes were observed only for coordinate b* (Table 5), which ranged from 14 to 20, presenting the highest values (*ca.* 19–20, more yellow) in Grécia and Restauração.

In wholemeal flour, L* values were higher than in grain, and ranged from 80–88 (Table 5). Grécia and Restauração showed the highest values under control. Heat increased L* in Grécia (4%) and Nabão (2%). As regards coordinates a* and b*, differences between genotypes were observed only for coordinate b* (Table 5), which ranged from 9 to 13 both under control and heat conditions. Heat increased b* (15%) in T94.

### 2.6. Principal Component Analysis of Grain Composition and Quality Parameters for Control and Heat Treatments

**Figure 2:**
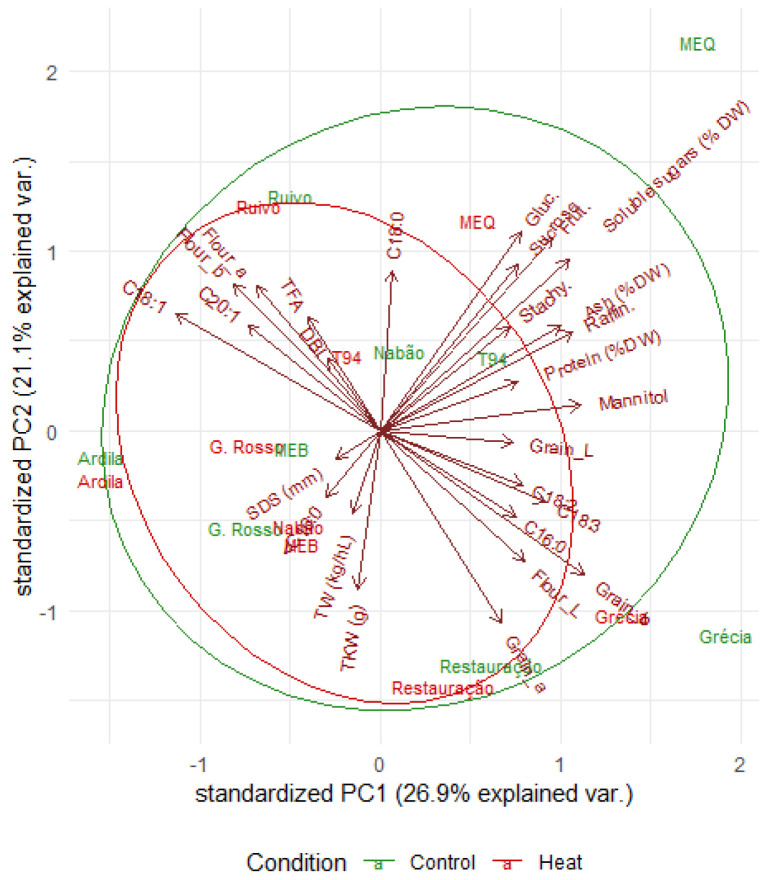
The principal component analysis plot for PC1 and PC2 based on the trait means. Trait vectors displaying angles smaller than 90° have positive association, while those with angles greater than 90° have negative association.

Principal component analysis was performed for the 19 parameters under study in both control and heat treatment conditions. Principal component one (PC1) contributed 26.94% for the total variation and principal component two contributed 21.09%, with a cumulative proportion of a 48% (Table 6). Variation among genotypes in principal component one was mainly attributed to the grains’ soluble sugars, fructose, and mannitol, as well as C18:1, grain ash, and grain_b. On the other hand, the observed variation in PC2 was mainly correlated with grain_a, soluble sugars, flour_a and flour_b, C18:0, sucrose, glucose, and fructose as plotted in Figure 2. Principal component three (PC3) accounted for 15.08% of the total variation, which mainly reflects the contributions of stachyose, TKW, fatty acids (<0.5% and C20:1), TFA, and DBI parameters. Altogether, the three main principal components show a cumulative variance of 63.1% (Table 6).

## 3. Discussion

Flour yield was evaluated through hectoliter weight, or test weight (TW), which corresponds to the mass of 100 liters expressed in kilograms. This parameter is widely used as a traditional marketing measure [27]. It indirectly expresses quality of grain, giving information on intrinsic features related to milling—namely grain shape, texture of seed coat (integument), grain size, and weight [27]. The higher the TW value, the greater the market acceptance and valuation of the product [18]. It is therefore considered an indicator of flour-yield potential, which is greatly determined by climatic parameters, particularly high temperature during the grain filling. According to [28], wheat may be considered as very heavy (TW 80–83 Kg/hL) or heavy (TW 76–79 Kg/hL). In this study, under nonstress conditions, some genetic variability occurred as concerns with TW, although only Ardila presented values in the range of heavy wheat (*ca.* 78 Kg/hL). Despite a heat-induced TW decrease, Ardila remained amongst the genotypes presenting the highest values under stress. TW lowering may occur under unfavorable conditions, reflecting grain weight reduction and less available endosperm to obtain flour, and the resulting grain would be classified as unsuitable for bread production [29]. This was not the case in Nabão, Grécia, and Restauração, where TW significantly increased with heat, suggesting an improved flour-yield potential of these varieties under high temperature.

Thousand-kernel weight (TKW) is mainly related to the size and density of the grain, but it also depends on the variety and environmental conditions (filling and maturation of the grain) [30]. During early seed development, cell proliferation is controlled by seed-size regulatory genes, which are regulated by abiotic stresses [30,31]. Heat may affect some grain-yield components, such as TKW, although some negative impacts may be attenuated by preanthesis stress [32]. Smaller grain size (lower TKW) may occur concomitantly to unaltered TW, suggesting the formation of well-shaped kernels and preservation of grain filling, which was the case in MEQ. In terms of the milling industry, the sieving of smaller grains implies larger grain waste, and may result in flour-yield reductions [27]. MEB showed considerably higher TKW under heat, as well as T94.

In cereals, carbohydrates supporting grain growth during the filling stage are derived from the current photo-assimilate and stored carbohydrate reserves in vegetative organs, which can be remobilized into the grains [3,32], suggesting that similar mechanisms may be present in varieties showing stable or increased grain production under postanthesis heat. Results for Nabão (higher TW), T94 and MEB (higher TKW) are consistent with increased grain production (g/plant), previously reported under high temperature [12]. Grain-yield reduction under heat may derive from inhibition of starch synthase, decreasing the conversion of sucrose into starch, or enhanced respiration leading to lowering of grain sugars and other stored compounds [3,4,15,16,33].

As regards soluble sugars, according to [6] concentrations of total sugars during grain filling were not significantly affected by high temperatures in bread and durum wheat. However, the authors reported some intraspecific genetic variability in levels of reducing sugars and suggested their involvement in tolerance signaling, possibly developmentally regulated, modulated by the sugar pool. Overexpression of sucrose transporter HvSUT1 increases grain protein content in *T. aestivum*, but also upregulates gene expression of positive and negative regulators related to sugar signaling and assimilate supply [34], highlighting sugar’s physiological role in the adjustment ability of the grain storage metabolism in response to metabolic alterations.

In this work, sucrose values obtained for bread-wheat kernels were similar to those described in the literature [35], although some variation was observed not only between genotypes, but also as a result of postanthesis heat. Heat induced an increase of total soluble sugars in Gentil Rosso, mainly due to augmented sucrose. The MEQ variety presented the highest values of the most abundant sugars (sucrose, raffinose, and stachyose) in control and heat-stress plants, despite heat-induced total sugars decreasing. Nonstarch endogenous sugars may have beneficial effects for the bread-making industry, contributing as substrate in the yeast fermentation process [19]. Yeast cells consume the fermentable sugars present in dough and generate carbon dioxide (CO_2_) and ethanol that are responsible for dough leavening during the fermentation phase and the oven rise. In the germplasm under study, control plants presented higher values of the trisaccharide raffinose (galactose, glucose, and fructose units) than reported in the literature (0.19–0.68% of DW) [35], with Ardila showing the lowest and MEQ the highest values (0.68 and 1.49%, respectively). Such a distinct range of values probably depends on the genetic backgrounds of the ancient varieties under study. Raffinose, together with fructans, belongs to a group of small fermentable carbohydrates termed FODMAPs (fermentable, oligo-, di-, and monosaccharides, and polyols). Wheats containing less raffinose could be of interest for developing low-FODMAP food products, since it has been suggested that a low-FODMAP diet improves the management of irritable bowel syndrome (IBS) and inflammatory bowel disease (Crohn’s disease and ulcerative colitis), by reducing fermentation in the colon [36].

Nitrogen accumulation in the grain is less negatively affected by high temperature than carbohydrate accumulation, because most plant nitrogen uptake generally occurs before anthesis [37]. Breeding programs are promoting the increase of protein content and quality characteristics, both in bread and durum wheat, in order to obtain better flour and semolina parameters to satisfy the needs of the industry and the consumers [38]. In this study, genotypes in control conditions presented some diversity in protein content, which varied between 20.4% (T94) and 17.0% (Nabão), in accordance with values reported for *T. aestivum* [6]. All these protein values are above national reference values [17], which probably reflects the said breeding effort. Under heat, protein content decreased or was unaltered in all genotypes except in Ardila, which stood out for its heat-induced protein increase. In *T. aestivum*, bread-making quality depends on the amount and composition of gluten proteins in the endosperm, which determine the viscoelasticity properties of dough, evaluated through SDS [18]. This quality feature can be affected by high temperatures during grain filling [4,6]. In the presently studied germplasm, high SDS values (>45 mm) were found in the control plants of Gentil Rosso, Restauração, Nabão and MEB, indicating a higher gluten strength and probably the obtention of more suitable flours for bread-making purposes. SDS was unaltered by heat in the majority of genotypes except in T94, denoting unaffected gluten strength and wheat-flour quality under stress. Other authors reported heat-induced changes in levels of essential amino acids during grain filling, which might affect gluten strength, hence diminishing the wheat-flour quality [6]. The increase in SDS observed in T94 as a result of heat indicates a better dough elasticity, and a higher suitability for bread making.

The color of the grain/flour is an important rheological feature for assessing the quality of wheat grains, since it reflects the content of carotenoids, proteins, fibers, and the presence of impurities in the grind [9,38]. Lightness (100-white and zero–black) is influenced by the content of bran and other foreign (impurities) materials in the flour and is directly related to the ash content. Chromaticity coordinates a* (positive values—redness or negative values—greenness) and b* (positive values—yellowness or negative values—blueness) are the result of the presence of natural pigments, and are usually more important for durum wheat evaluation [9,38]. Color variability may be used to match specific industry requirements related to grain end-use products, namely for the bread-making and pasta industry. As expected, L values were lower in grain than in flour. Little variation was found between genotypes regarding ash content or grain and flour color parameters. The few heat-induced changes occurred in grain lightness (Gentil Rosso, Ruivo and MEQ) were not reflected in flour values. In flour, L decreases observed in Grécia and Nabão may be related to their lower ash content under heat.

Wheat kernels also contain relatively small quantities of lipids, usually from 2 to 3.5%, which nevertheless play an important role in cereal processing and the nature of grain products by affecting the properties of protein and starch [1,14]. Under control conditions, genotype variability was present in genotypes’ total fatty acids (TFA) amounts, which expresses lipids content. The highest values (14–15 mg g^−1^ DW) were observed in T94, Ruivo, and Ardila. Endogenous nonstarch lipids tremendously affect fresh bread quality, being key players in bread leavening and crumb structure, through their impact on gas cell stability by influencing the strength of the gluten–starch matrix in dough, which supports expanding gas cells [39]. Also, lipids are thought to have a direct impact on gas cell stability by being present as surface-active agents in the liquid films that surround expanding gas cells in dough and act as a secondary stabilization mechanism. Both impact mechanisms are in play during the mixing, fermentation, proofing, and/or first baking stages [39].

Studies in *T. aestivum* have shown that growth at elevated temperatures reduced the amounts of accumulated lipids, particularly nonpolar lipids [21]. Significant changes in the acyl composition of individual lipids were also observed, most often in the proportions of palmitate (C16:0), oleate (C18:1), and linoleate (C18:2). The observed alterations in wheat lipids are likely to affect the properties of any flours derived from grain grown under climate change conditions [21].

Also, the sowing date may interact with cultivar traits, especially in the case of total lipids and some fatty acids (FAs) content [22]. The present results highlight that lipid content under heat was maintained in all genotypes except Ardila, suggesting it may be a relevant trait under high temperature. Stability was present also as regards the FAs profile (C18:2 > C16:0 > C18:1 > C18:3 > C18:0 > C20:1), that was preserved under stress. In spite of this fact, some changes in the relative abundance of FAs should be highlighted, namely the increases of polyunsaturated C18:2 and monounsaturated C18:1 in Grécia under heat. FAs in wheat lipids are mostly polyunsaturated, which increases their value for human nutrition [23,24]. Wheat grains are valuable sources of essential FAs (linoleate and α-linolenate), that are fundamental in the human diet. A high proportion of unsaturated FAs compared to saturated ones in food protects against obesity, cardiovascular diseases, and inflammatory processes [1,23]. Variation in lipids and their FAs of Italian durum wheat cultivars was used to set proper quality standards for wholegrain flours and products where the germ should be preserved, considering also the recent interest of industry and consumers for these kind of products [24].

The major lipids in wheat kernels are nonpolar lipids, consisting of more abundant triacylglycerols (TAGs), and lower levels of monoacylglycerols (MAGs), diacylglycerols (DAGs), and free fatty acids (FFAs). These nonpolar storage lipids are typically found in oil bodies or spherosomes in the germ and aleurone tissues. Wheatgerm oil is one of the most important commercial germ oils [14]. Nonstarch endosperm lipids contain a fraction (33.2% to 47.4%) of nonpolar lipids (predominantly TAGs originating from oil bodies located in subaleurone and endosperm cells), and a comparable portion of polar lipids (galactolipids and phospholipids) which are remainders of lipid bilayers surrounding amyloplasts. Starch lipids are located inside the starch granules and consist almost exclusively (89% to 94%) of phospholipids [40]. Lipids are unevenly distributed over the different structural parts of the grain. About 35% to 45% occur in the endosperm, 30% to 36% in the germ, 25% to 29% in the aleurone, and only a minor portion in the pericarp (<4%) [1]. Upon wheat milling into flour, kernels and kernel pieces are broken, further reduced in size and sieved to separate the endosperm from the bran (i.e., pericarp and aleurone) and the germ [40]. Lipids in the resultant flour are not only those of the endosperm, but they also include a portion of those of the aleurone and germ. In wholemeal flour, lipases are more abundant due to their presence in seed coat (integument), increasing the risk of lipoperoxidation [1,20]. Under control conditions, varieties showed similar lipid unsaturation, expressed as DBI index. Overall lipid unsaturation was maintained under stress, which could constitute a strong benefit attending to the fact that oxidation of the polyunsaturated acids can lead to off-flavors during storage [20].

Over the centuries of genetic manipulation, certain characters have been privileged, but others have been lost, particularly in the field of lipids: the content of unsaturated fatty acids has decreased by half or even a third. A reduction in unsaturated fatty acids was associated with selection during primary *Triticum* domestication [25,26]. The level and composition of wheat lipids depend on the growing environment and the genetic background of the wheat, among other factors [1]. Wheat-flour lipids indeed play important roles during bread making, and therefore, have great potential as targets for improving bread quality [1]. Nowadays there is also an increasing in elucidating the metabolism of storage lipids, particularly in cereal grains, with the scope to redirect carbon from starch to oil in the endosperm, in the face of an increasing demand for vegetable oils as competitive alternatives to mineral hydrocarbon-based products [41].

In the present study, the differentiation of grains into different clusters was due to relatively high contribution of few parameters rather than small contribution from each character. Considering the conditions studied, some varieties clustered in approximate positions regardless of the treatment (control or heat). In fact, Ardila, Restauração, and Ruivo seem to be less susceptible to heat during grain filling, meaning that they are well-adapted to the high temperatures that are frequent in the region where they are typically cultivated. In contrast, MEQ seems to be more differentially affected during grain filling.

All results were used for genetic diversity evaluation of the physiological answer to heat and respective impact on grain filling and quality. This knowledge may be used for breeding design, aiming for an improvement of wheat physiological adaptation to heat, in the scenario of predicted climate changes.

## 4. Materials and Methods

### 4.1. Plant Material and Experimental Design

**Figure 3:**
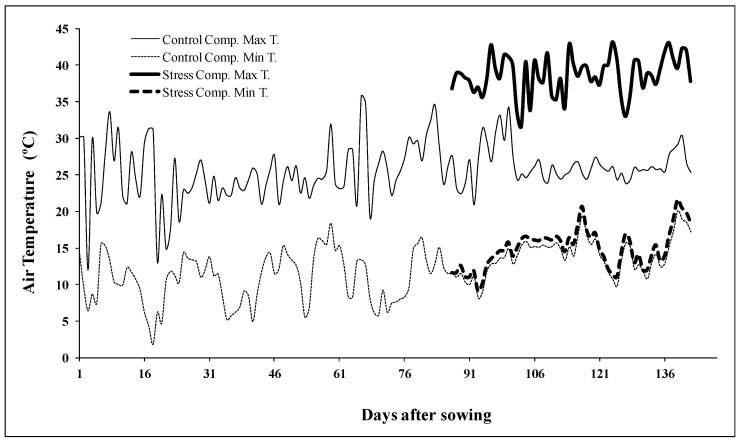
Maximal and minimal diurnal temperatures in two compartments of a semi-controlled greenhouse during the wheat cycle. Day 1 corresponds to the sowing date (23 December).

Germplasm consisted of seven ancient landraces and traditional varieties from a Portuguese wheat collection [42] preserved in INIAV: Gentil Rosso, Grécia, Mocho de Espiga Branca (MEB), Mocho de Espiga Quadrada (MEQ), Transmontano 94 (T94), Restauração, and Ruivo. Two commercial varieties (Ardila and Nabão) were also included in the study. Plants were grown in a semicontrolled greenhouse, according to the experimental design described in [12]. Growth conditions were monitored through data loggers (Mezão Lda, Portugal). Natural irradiation ranged from 400–1000 µmol m^−2^ s^−1^. Ten days after visual assessment of anthesis, which corresponded to the grain-filling stage and varied according to genotype, half of the pots were left in control conditions (maximal diurnal temperature *ca.* 23–26 °C), and the other half was placed in another compartment to be subjected to heat stress (*ca.* 10 °C higher maximal temperatures as observed in Figure 3). After heat imposition (*ca.* 7 days). plants returned to control conditions. All plants were well-watered during the whole life cycle. At full maturation shoots were harvested and oven dried for 72 h at 35 °C. Spikes were threshed manually.

Nutritional and quality parameters were evaluated using three to six replications for each genotype and treatment.

### 4.2. Test Weight (TW), Thousand-Kernel Weight (TKW), and Obtention of Wholemeal Flour

Test weight (TW) and thousand-kernel weight (TKW) were used to assess grain quality [9,27]. For estimation of TW, the volume of each sample (grain production per plant) was weighed, and results expressed in Kg/hL. TKW correspond to the weight of 1000 grain samples.

Grain samples of *ca.* 20 g were reduced to wholemeal flour using a blade mill (Polymix Culatti, Lucerne, Switzerland) equipped with a 1 mm mesh sieve. Flour samples were kept in a moisture-free atmosphere until analysis. 

### 4.3. Soluble Sugars Composition

Soluble sugars were extracted from flour samples (400 mg DW), following the method of [43]. Samples were homogenized in 20 mL of cold H_2_O, stirred for 30 min on ice, sonicated for 5 min and centrifuged (15,000 g, 20 min, 4 °C). The supernatant was collected, and extraction procedure was repeated with the pellet. Both supernatants were joined and cleared with nylon syringe filters (0.45 µm) before injection. Sugars separation was performed with an HPLC system coupled to a refractive index detector (Model 2414, Waters, MA, USA), using a SugarPak 1 column (300 mm length × 6.5 mm diameter, Waters) at 90 °C, with H_2_O as eluent (containing 50 mg EDTA-Ca L^−1^ H_2_O) and a flow rate of 0.5 mL min^−1^. Standard curves were used for the quantification of each sugar.

### 4.4. Lipid Profile

Total lipids were extracted from flour samples (1 g DW), as described by [44]. Extraction was performed with 20 mL of *n*-hexane while stirring, at room temperature, for 15 min. After centrifugation (4500× g, 10 min, 15 °C) the solvent was collected, and extraction procedure was repeated with the pellet. Both supernatants were joined, evaporated to dryness under N_2_ flow and resuspended in ethanol:toluene (1:4, *v*/*v*). Aliquots of 50 µL of the samples were saponified and methylated with BF_3_–methanol (Merck, Kenilworth, NJ, USA), using heptadecanoic acid (C17:0) as internal standard. The fatty acid methyl esters were analyzed with a GC-FID chromatograph (CP-3380, Varian, Palo Alto, CA, USA). Separation was performed using a fused silica capillary column (DB-WAX, 0.25 mm i.d. × 30 m, 0.25 µm; J&W Scientific, Folsom, CA, USA). Column temperature was programmed to rise from 80 °C to 200 °C at 12 °C min^–1^ after 2 min at the initial temperature. Injector and detector temperatures were 200 °C and 250 °C, respectively. Carrier gas was hydrogen with a flow rate of 1 mL min^–1^, at a split ratio of 1:100 of the sample, as described in [12]. Individual fatty acids (FAs) were identified by comparison with known Sigma and Supelco standards. The most representative Fas (>0.5%) under control conditions were quantified, namely palmitic (C16:0), stearic (C18:0), oleic (C18:1), linoleic (C18:2), linolenic (C18:3), and gadoleic (C20:1) acids. Total fatty acids (TFA) is the sum of individual FAs. The degree of unsaturation of TFA was obtained through a double bond index (DBI), calculated according to the formula: DBI = (% monoenes + 2 × % dienes + 3 × % trienes)/% saturated FAs [45].

### 4.5. Protein Content, SDS Sedimentation Test, and Ash Content

Protein amounts were quantified from flour samples (1 g DW) according to the Kjeldahl method [46] by adding 12.5 mL of sulfuric acid (95–97%), using potassium sulfate and selenium as catalyzers. Digestion took place at *ca.* 420 °C for 2 h. After cooling the samples at ambient temperature, distillation was performed to remove ammonia. Boric acid (4% *w*/*v*) and some drops of methyl red were added to 150 mL of the distilled liquid, prior to titration with hydrochloric acid 0.1 N.

The sodium dodecyl sulfate (SDS) sedimentation test was performed on whole-flour samples (1 g DW), according to [47]. This test is considered a rapid method to estimate gluten strength and proteins quality. 

Ash contents were determined from flour samples (5 g DW) according to [48] method. After incineration at 900 °C the samples were maintained in a desiccator prior to weighing. Results were expressed as ash percentage per DW.

### 4.6. Color Measurements

The flour color parameters, lightness (L*) and chromaticity (coordinates a* and b*), were obtained with a Minolta CR 300 colorimeter (Minolta Corp., Ramsey, NJ, USA) coupled with a glass container for solid samples (CR-A504). Measurements were performed for illuminant D_65_ based on CIE (Commission Internationale de l’Éclairage) L*a*b* system. Color evaluation was also performed in whole-grain samples.

### 4.7. Statistical Analysis

A two-way ANOVA (*p* < 0.05) was applied, using Statistix 9 (Analytical Software, 2009), followed by a Tukey test for mean comparison (95% confidence level). In figures and tables different letters express significantly different results between genotypes for each treatment (a, b, c, d, e) or between control and heat treatment for the same genotype (r, s), a and r representing the highest values.

PCA analysis for 19 parameters was performed in R v4.1.2 using the built-in “stats” package [49], and data from the two main principal components was plotted using the “ggbiplot” package v 0.55 [50].

## 5. Conclusions

The present work was undertaken to evaluate the nutritional and quality traits of Portuguese bread wheat germplasm, including ancient landraces and commercial varieties, under high-temperature conditions after anthesis (the filling stage), which frequently occurs in the natural environment. Genetic diversity is scarce as regards heat tolerance, and wheat productivity and quality in warmer climates are still major concerns for breeders in Mediterranean region. Our results highlighted some variability in germplasm under study as regards grain nutritional and quality features both under control and heat conditions. Some traits were negatively affected, but some others were unaltered or even improved under stress. Cluster analysis of traits indicated a lower susceptibility to heat in Ardila, Restauração, and Ruivo, reflecting a better adaptation to high temperatures in contrast to MEQ, which seems to be more differentially affected during grain filling.

The characterization and identification of more favorable features under adverse environments may be relevant for agronomic, industrial, or breeding purposes, in view of a better crop adaptation to changing climate and an improved crop sustainability in agricultural systems more prone to heat stress. This information contributes for characterization and selection of genotypes that may be used by breeders as parental lines for crossings and obtention of new varieties more adapted to changing climate. This is an important aim of the Portuguese Cereal Breeding Program.

Genotypes presenting interesting nutritional traits such as protein, sugars, and FAs, might be further explored as a potential source of health-beneficial food products viewing new markets emerging from integrated farming systems.

## Figures and Tables

**Table 1:** Evaluation of test weight (TW) and thousand-kernel weight (TKW) in nine bread-wheat genotypes under control conditions (Ctr) or subjected to heat after anthesis. Mean values ± SE (*n* = 3). Different letters express significant differences between genotypes for each treatment (^a^, ^b^, ^c^, ^d^, ^e^) or between control and heat treatment for the same genotype (^r^, ^s^); ^a^ and ^r^ represent the highest values.

Genotypes	Treatment	TW (kg/hL)	TKW (g)
Ardila	Ctr	77.89 ± 0.65 ^a r^	38.23 ± 0.61 ^b r^
	Heat	75.00 ± 1.24 ^ab s^	33.57 ± 0.08 ^bc s^
G. Rosso	Ctr	69.79 ± 0.63 ^cd r^	46.27 ± 0.84 ^a r^
	Heat	71.00 ± 0.89 ^bc r^	46.57 ± 0.12 ^a r^
Grécia	Ctr	74.13 ± 0.07 ^ab s^	33.57 ± 0.66 ^b r^
	Heat	77.62 ± 0.70 ^a r^	36.50 ± 0.07 ^b r^
MEB	Ctr	66.45 ± 1.13 ^d r^	25.01 ± 1.38 ^d s^
	Heat	63.27 ± 0.48 ^d s^	37.95 ± 0.96 ^b r^
MEQ	Ctr	69.00 ± 0.28 ^cd r^	32.13 ± 3.31 ^c r^
	Heat	67.69 ± 0.95 ^c r^	23.70 ± 1.68 ^d s^
Nabão	Ctr	71.86 ± 1.75 ^b s^	25.37 ± 0.35 ^d r^
	Heat	77.66 ± 1.58 ^a r^	28.63 ± 0.28 ^cd r^
Restauração	Ctr	71.51 ± 0.54 ^b s^	37.52 ± 0.78 ^bc r^
	Heat	74.32 ± 0.44 ^ab r^	38.10 ± 0.21 ^b r^
Ruivo	Ctr	72.89 ± 0.32 ^bc r^	18.45 ± 2.48 ^e r^
	Heat	72.49 ± 0.14 ^b r^	18.92 ± 0.85 ^e r^
T94	Ctr	62.62 ± 1.15 ^d r^	18.87 ± 0.33 ^e s^
	Heat	64.37 ± 0.16 ^d r^	25.74 ± 0.68 ^d r^

**Table 2:** Soluble sugars (% DW) in wholemeal flour obtained from grains of nine bread-wheat genotypes under control conditions (Ctr) or subjected to heat after anthesis. Mean values ± SE (*n* = 6). Different letters express significant differences between genotypes for each treatment (^a^, ^b^, ^c^, ^d^, ^e^) or between control and heat treatment for the same genotype (^r^, ^s^); ^a^ and ^r^ represent the highest values.

Genotype	Treat.	Stachy.	Raffin.	Sucrose	Gluc.	Frut.	Mannitol	Total
Ardila	Ctr	0.21 ± 0.00 ^c s^	0.68 ± 0.01 ^e r^	1.51 ± 0.03 ^cd r^	0.13 ± 0.01 ^e r^	0.09 ± 0.00 ^c r^	0.04 ± 0.00 ^cd r^	2.66 ± 0.05 ^e r^
	Heat	0.24 ± 0.01 ^b r^	0.76 ± 0.01 ^e r^	1.44 ± 0.02 ^d r^	0.14 ± 0.00 ^c r^	0.07 ± 0.01 ^c s^	0.04 ± 0.00 ^b r^	2.70 ± 0.04 ^d r^
G. Rosso	Ctr	0.30 ± 0.01 ^b r^	1.00 ± 0.02 ^cd r^	1.33 ± 0.02 ^d s^	0.17 ± 0.01 ^de r^	0.10 ± 0.01 ^c r^	0.03 ± 0.00 ^d s^	2.93 ± 0.04 ^de s^
	Heat	0.30 ± 0.01 ^a r^	0.96 ± 0.02 ^cd s^	1.56 ± 0.04 ^cd r^	0.17 ± 0.01 ^bc r^	0.11 ± 0.01 ^c r^	0.05 ± 0.00 ^b r^	3.15 ± 0.07 ^c r^
Grécia	Ctr	0.29 ± 0.01 ^b r^	1.10 ± 0.03 ^bc r^	1.85 ± 0.04 ^b r^	0.18 ± 0.01 ^cd s^	0.15 ± 0.02 ^bc r^	0.07 ± 0.00 ^a r^	3.65 ± 0.04 ^b r^
	Heat	0.28 ± 0.00 ^ab r^	1.01 ± 0.03 ^b s^	1.86 ± 0.03 ^a r^	0.21 ± 0.02 ^ab r^	0.15 ± 0.02 ^ab r^	0.07 ± 0.00 ^a r^	3.58 ± 0.05 ^ab r^
MEB	Ctr	0.22 ± 0.00 ^c r^	0.94 ± 0.05 ^d r^	1.64 ± 0.04 ^c r^	0.20 ± 0.01 ^cd r^	0.14 ± 0.02 ^bc r^	0.04 ± 0.00 ^cd r^	3.18 ± 0.07 ^cd r^
	Heat	0.20 ± 0.02 ^c s^	0.92 ± 0.03 ^d r^	1.67 ± 0.06 ^bc r^	0.18 ± 0.01 ^bc s^	0.12 ± 0.02 ^bc r^	0.04 ± 0.00 ^b r^	3.13 ± 0.12 ^c r^
MEQ	Ctr	0.42 ± 0.02 ^a r^	1.49 ± 0.08 ^a r^	2.22 ± 0.03 ^a r^	0.38 ± 0.01 ^a r^	0.32 ± 0.01 ^a r^	0.06 ± 0.00 ^ab r^	4.89 ± 0.09 ^a r^
	Heat	0.32 ± 0.01 ^a s^	1.26 ± 0.03 ^a s^	1.82 ± 0.06 ^ab s^	0.21 ± 0.01 ^ab s^	0.20 ± 0.04 ^a s^	0.05 ± 0.00 ^b r^	3.86 ± 0.06 ^a r^
Nabão	Ctr	0.24 ± 0.01 ^c r^	1.23 ± 0.03 ^b r^	1.82 ± 0.04 ^b r^	0.20 ± 0.01 ^cd r^	0.16 ± 0.01 ^b r^	0.05 ± 0.00 ^bc r^	3.71 ± 0.08 ^b r^
	Heat	0.19 ± 0.00 ^c s^	1.15 ± 0.02 ^ab r^	1.58 ± 0.04 ^cd s^	0.14 ± 0.01 ^c s^	0.13 ± 0.01 ^b s^	0.05 ± 0.00 ^b r^	3.24 ± 0.06 ^c s^
Restauração	Ctr	0.22 ± 0.01 ^c r^	1.09 ± 0.02 ^cd r^	1.48 ± 0.03 ^cd r^	0.15 ± 0.00 ^e r^	0.13 ± 0.00 ^bc r^	0.06 ± 0.01 ^ab r^	3.13 ± 0.06 ^cd r^
	Heat	0.20 ± 0.01 ^cr^	1.12 ± 0.02 ^ab r^	1.46 ± 0.06 ^c r^	0.16 ± 0.01 ^c r^	0.12 ± 0.01 ^b r^	0.04 ± 0.00 ^b s^	3.10 ± 0.09 ^c r^
Ruivo	Ctr	0.20 ± 0.01 ^c s^	0.93 ± 0.02 ^d s^	2.18 ± 0.04 ^a r^	0.22 ± 0.01 ^bc r^	0.17 ± 0.01 ^b r^	0.06 ± 0.00 ^ab r^	3.76 ± 0.03 ^b r^
	Heat	0.25 ± 0.01 ^b r^	1.08 ± 0.02 ^bc r^	1.73 ± 0.04 a^bc s^	0.24 ± 0.01 ^a r^	0.18 ± 0.01 ^ab r^	0.05 ± 0.00 ^b r^	3.54 ± 0.05 ^b s^
T94	Ctr	0.22 ± 0.01 ^c r^	1.04 ± 0.02 ^cd r^	1.55 ± 0.02 ^c r^	0.26 ± 0.01 ^b r^	0.20 ± 0.01 ^b r^	0.06 ± 0.00 ^ab r^	3.33 ± 0.05 ^c r^
	Heat	0.16 ± 0.01 ^c s^	0.96 ± 0.01 ^cd r^	1.58 ± 0.02 ^cd r^	0.24 ± 0.00 ^a r^	0.18 ± 0.00 ^ab r^	0.05 ± 0.00 ^b r^	3.18 ± 0.03 ^c r^

**Table 3:** Protein, gluten strength (SDS) and ash content in wholemeal flour obtained from grains of nine bread wheat genotypes under control conditions (Ctr) or subjected to heat after anthesis. Mean values ± SE (*n* = 3). Different letters express significant differences between genotypes for each treatment (^a^, ^b^, ^c^, ^d^) or between control and heat treatment for the same genotype (^r^, ^s^); ^a^ and ^r^ represent the highest values.

Genotypes	Treatment	Protein (% DW)	SDS (mm)	Ash (% DW)
Ardila	Ctr	17.20 ± 0.21 ^c s^	29.67 ± 0.33 ^c r^	2.16 ± 0.15 ^ab r^
	Heat	18.23 ± 0.23 ^ab r^	30.00 ± 0.00 ^c r^	1.91 ± 0.13 ^b s^
G. Rosso	Ctr	18.97 ± 12 ^b r^	53.00 ± 0.58 ^a r^	1.89 ± 0.13 ^b r^
	Heat	17.37 ± 0.67 ^b s^	52.00 ± 1.00 ^a r^	1.96 ± 0.05 ^ab r^
Grécia	Ctr	20.03 ± 0.07 ^ab r^	22.00 ± 2.65 ^d r^	2.37 ± 0.07 ^a r^
	Heat	17.60 ± 0.17 ^b s^	24.33 ± 2.19 ^c r^	2.10 ± 0.04 ^ab s^
MEB	Ctr	16.97 ± 0.03 ^c r^	45.00 ± 1.15 ^ab r^	2.13 ± 0.06 ^ab r^
	Heat	16.65 ± 0.14 ^c r^	47.50 ± 0.29 ^ab r^	2.04 ± 0.04 ^ab r^
MEQ	Ctr	19.37 ± 0.33 ^a r^	41.67 ± 1.86 ^bc r^	2.41 ± 0.08 ^a r^
	Heat	19.20 ± 0.10 ^a r^	43.67 ± 1.86 ^b r^	2.33 ± 0.02 ^a r^
Nabão	Ctr	16.97 ± 0.18 ^c r^	51.67 ± 0.88 ^a r^	2.15 ± 0.08 ^ab r^
	Heat	16.87 ± 0.28 ^c r^	53.67 ± 1.45 ^a r^	1.81 ± 0.13 ^b s^
Restauração	Ctr	17.20 ± 0.06 ^c r^	52.331.45 ± ^a r^	2.15 ± 0.04 ^ab r^
	Heat	16.97 ± 0.12 ^c r^	52.00 ± 0.58 ^a r^	2.10 ± 0.09 ^ab r^
Ruivo	Ctr	17.47 ± 0.09 ^c r^	34.00 ± 1.73 ^c r^	2.15 ± 0.05 ^ab r^
	Heat	16.40 ± 0.25 ^c s^	31.33 ± 4.37 ^c r^	2.12 ± 0.08 ^ab r^
T94	Ctr	20.35 ± 0.14 ^a r^	36.50 ± 2.02 ^c s^	2.14 ± 0.07 ^ab r^
	Heat	17.63 ± 0.15 ^b s^	43.00 ± 1.15 ^b r^	2.11 ± 0.04 ^ab r^

**Table 4:** Fatty acids composition (mol%) and total fatty acids unsaturation, estimated through a double bond index (DBI), in wholemeal flour obtained from grains of nine bread-wheat genotypes under control conditions (Ctr) or subjected to heat after anthesis. Mean values ± SE (*n* = 3). Different letters express significant differences between genotypes for each treatment (^a^, ^b^, ^c^, ^d^, ^e^) or between control and heat treatment for the same genotype (^r^, ^s^); ^a^ and ^r^ represent the highest values.

Genotype	Treat.	C16:0	C18:0	C18:1	C18:2	C18:3	C20:1	<0.5%	DBI
		mol (%)							
Ardila	Ctr	18.27 ± 0.13 ^b r^	0.96 ± 0.02 ^bc r^	15.80 ± 0.05 ^ab r^	57.83 ± 0.17 ^abc r^	4.06 ± 0.07 ^b r^	0.79 ± 0.01 ^b r^	2.25 ± 0.14 ^a r^	6.86 ± 0.09 ^a r^
	Heat	19.80 ± 0.64 ^a r^	0.93 ± 0.03 ^c r^	15.85 ± 0.12 ^b r^	56.40 ± 0.44 ^ab s^	3.86 ± 0.04 ^c r^	0.79 ± 0.01 ^a r^	2.36 ± 0.15 ^b r^	6.32 ± 0.22 ^a r^
G. Rosso	Ctr	21.47 ± 0.89 ^ab r^	1.06 ± 0.05 ^a s^	14.20 ± 0.28 ^cd s^	56.36 ± 0.82 ^bc r^	3.84 ± 0.06 ^b r^	0.63 ± 0.06 ^c r^	2.44 ± 0.39 ^a s^	5.88 ± 0.30 ^a r^
	Heat	19.32 ± 0.49 ^a r^	1.26 ± 0.08 ^b r^	15.54 ± 0.23 ^bc r^	55.62 ± 0.62 ^b s^	3.70 ± 0.24 ^c r^	0.66 ± 0.04 ^bc r^	3.90 ± 0.40 ^a r^	5.95 ± 0.20 ^a r^
Grécia	Ctr	23.20 ± 1.11 ^a r^	0.86 ± 0.03 ^c r^	11.16 ± 0.53 ^f s^	57.11 ± 1.41 ^abc s^	4.76 ± 0.21 ^a r^	0.62 ± 0.06 ^c r^	2.30 ± 0.20 ^a r^	5.58 ± 0.37 ^a s^
	Heat	19.63 ± 0.56 ^a s^	0.97 ± 0.01 ^c r^	12.20 ± 0.17 ^e r^	59.54 ± 0.63 ^a r^	4.53 ± 0.06 ^a r^	0.61 ± 0.04 ^c r^	2.52 ± 0.47 ^b r^	6.53 ± 0.28 ^a r^
MEB	Ctr	20.70 ± 1.00 ^ab r^	1.06 ± 0.06 ^a r^	14.74 ± 0.26 ^bc r^	56.30 ± 0.80 ^bc r^	3.82 ± 0.07 ^b r^	0.87 ± 0.05 ^ab r^	2.51 ± 0.21 ^a r^	6.02 ± 0.37 ^a r^
	Heat	20.96 ± 1.13 ^a r^	0.96 ± 0.02 ^c r^	14.36 ± 0.22 ^bcd r^	56.91 ± 0.61 ^ab r^	3.88 ± 0.05 ^c r^	0.85 ± 0.06 ^ab r^	2.07 ± 0.27 ^bc r^	6.07 ± 0.30 ^a r^
MEQ	Ctr	20.70 ± 1.07 ^ab r^	1.23 ± 0.02 ^a s^	13.27 ± 0.24 ^cde r^	58.40 ± 0.74 ^abc r^	4.12 ± 0.07 ^b r^	0.59 ± 0.04 ^c r^	1.69 ± 0.15 ^ab r^	6.27 ± 0.34 ^a r^
	Heat	19.60 ± 1.03 ^a r^	1.53 ± 0.03 ^a r^	14.14 ± 0.22 ^cd r^	58.59 ± 0.75 ^ab r^	3.98 ± 0.07 ^b r^	0.62 ± 0.05 ^c r^	1.50 ± 0.15 ^c r^	6.56 ± 0.35 ^a r^
Nabão	Ctr	21.00 ± 0.90 ^ab r^	1.10 ± 0.04 ^ab r^	13.03 ± 0.19 ^de r^	57.81 ± 0.64 ^abc r^	4.62 ± 0.08 ^a r^	0.89 ± 0.07 ^ab r^	1.55 ± 0.08 ^ab r^	6.30 ± 0.33 ^a r^
	Heat	20.52 ± 1.15 ^a r^	1.00 ± 0.02 ^c r^	13.43 ± 0.22 ^de r^	58.38 ± 0.78 ^ab r^	4.40 ± 0.07 ^a r^	0.92 ± 0.07 ^a r^	1.35 ± 0.06 ^c r^	6.58 ± 0.41 ^a r^
Restauração	Ctr	20.01 ± 0.86 ^ab r^	0.95 ± 0.03 ^bc r^	13.01 ± 0.35 ^de r^	60.08 ± 0.87 ^a r^	4.13 ± 0.06 ^b r^	0.57 ± 0.04 ^c r^	1.26 ± 0.11 ^b r^	6.81 ± 0.35 ^a r^
	Heat	21.87 ± 1.13 ^a r^	0.96 ± 0.04 ^c r^	13.01 ± 0.68 ^de r^	58.05 ± 1.14 ^ab r^	4.35 ± 0.20 ^ab r^	0.60 ± 0.06 ^c r^	1.16 ± 0.09 ^c r^	6.17 ± 0.40 ^a r^
Ruivo	Ctr	20.07 ± 0.87 ^ab r^	1.06 ± 0.08 ^a r^	16.92 ± 0.72 ^a r^	55.69 ± 0.33 ^c r^	3.97 ± 0.06 ^b r^	1.09 ± 0.06 ^a r^	1.20 ± 0.04 ^b r^	6.56 ± 0.33 ^a r^
	Heat	19.68 ± 1.12 ^a r^	1.09 ± 0.02 ^bc r^	17.75 ± 0.27 ^a r^	55.46 ± 0.75 ^b r^	3.73 ± 0.06 ^c r^	1.02 ± 0.09 ^a r^	1.28 ± 0.08 ^c r^	6.66 ± 0.42 ^a r^
T94	Ctr	20.59 ± 0.64 ^ab r^	0.89 ± 0.02 ^c s^	12.56 ± 0.12 ^ef s^	59.35 ± 0.44 ^ab r^	4.55 ± 0.05 ^a r^	0.89 ± 0.04 ^ab r^	1.18 ± 0.02 ^b r^	6.63 ± 0.23 ^a r^
	Heat	19.88 ± 0.81 ^a r^	1.01 ± 0.02 ^c r^	13.57 ± 0.19 ^de r^	59.01 ± 0.54 ^ab r^	4.44 ± 0.05 ^a r^	0.90 ± 0.05 ^ab r^	1.19 ± 0.04 ^c r^	6.82 ± 0.29 ^a r^

DBI = [(% monoenes + 2 × % dienes + 3 × % trienes)/% saturated FAs].

**Table 5:** Color parameters (CIEL*a*b* system), determined through lightness (L*) and chromaticity coordinates (a* e b*), for grain and wholemeal flour of nine bread-wheat genotypes under control conditions (Ctr) or subjected to heat after anthesis. Mean values ± SE (Grain: *n* = 5; Flour: *n* = 3). Different letters express significant differences between genotypes for each treatment (^a^, ^b^, ^c^, ^d^, ^e^) or between control and heat treatment for the same genotype (^r^, ^s^); ^a^ and ^r^ represent the highest values.

		Grain			Flour		
**Genotype**	Treat.	L*	a*	b*	L*	a*	b*
Ardila	Ctr	51.93 ± 0.66 ^c r^	5.88 ± 0.19 ^a r^	14.43 ± 0.07 ^d r^	80.21 ± 0.17 ^e r^	2.77 ± 0.10 ^a r^	12.52 ± 0.12 ^a r^
	Heat	52.37 ± 0.16 ^e r^	5.81 ± 0.16 ^a r^	14.66 ± 0.16 ^d r^	79.38 ± 0.11 ^e r^	2.91 ± 0.03 ^a r^	12.61 ± 0.12 ^ab r^
G. Rosso	Ctr	55.80 ± 0.37 ^a s^	5.76 ± 0.19 ^a r^	16.18 ± 0.11 ^cd r^	84.59 ± 0.15 ^c r^	3.00 ± 0.04 ^a r^	12.78 ± 0.04 ^a r^
	Heat	59.88 ± 0.81 ^a r^	5.47 ± 0.14 ^a r^	16.33 ± 0.32 ^c r^	85.61 ± 0.46 ^b r^	3.06 ± 0.08 ^a r^	12.88 ± 0.07 ^a r^
Grécia	Ctr	57.02 ± 0.14 ^a r^	6.65 ± 0.11 ^a r^	20.32 ± 0.44 ^a r^	87.78 ± 0.46 ^a s^	1.51 ± 0.07 ^a r^	9.10 ± 0.21 ^b r^
	Heat	57.73 ± 0.36 ^b r^	6.39 ± 0.18 ^a r^	20.33 ± 0.16 ^a r^	90.99 ± 3.17 ^a r^	1.58 ± 0.06 ^a r^	9.14 ± 0.15 ^c r^
MEB	Ctr	53.95 ± 0.48 ^b r^	5.97 ± 0.20 ^a r^	16.02 ± 0.57 ^cd r^	84.43 ± 0.27 ^c r^	2.36 ± 0.09 ^a r^	10.66 ± 0.17 ^ab r^
	Heat	54.10 ± 0.74 ^d r^	5.65 ± 0.13 ^a r^	15.89 ± 0.57 ^cd r^	85.52 ± 0.49 ^bc r^	1.39 ± 0.12 ^a r^	9.97 ± 0.18 ^c r^
MEQ	Ctr	56.32 ± 0.29 ^a r^	5.60 ± 0.12 ^a r^	16.78 ± 0.27 ^c r^	81.86 ± 0.46 ^de r^	2.86 ± 0.15 ^a r^	12.30 ± 0.38 ^ab r^
	Heat	54.78 ± 0.56 ^cd s^	6.11 ± 0.14 ^a r^	16.22 ± 0.48 ^cd r^	82.23 ± 0.34 ^d r^	2.68 ± 0.10 ^a r^	11.89 ± 0.11 ^ab r^
Nabão	Ctr	54.14 ± 0.52 ^b r^	5.57 ± 0.08 ^a r^	15.14 ± 0.21 ^d r^	81.30 ± 0.65 ^de s^	2.56 ± 0.14 ^a r^	11.71 ± 0.77 ^ab r^
	Heat	53.12 ± 0.45 ^de r^	5.71 ± 0.16 ^a r^	15.25 ± 0.12 ^cd r^	83.24 ± 0.08 ^cd r^	2.02 ± 0.03 ^a r^	10.41 ± 0.13 ^bc r^
Restauração	Ctr	55.52 ± 0.47 ^a r^	7.01 ± 0.27 ^a r^	18.53 ± 0.37 ^ab r^	87.02 ± 0.57 ^ab r^	1.63 ± 0.14 ^a r^	9.19 ± 0.30 ^b r^
	Heat	55.32 ± 0.42 ^cd r^	6.89 ± 0.22 ^a r^	18.48 ± 0.35 ^ab r^	83.74 ± 0.41 ^b r^	1.59 ± 0.11 ^a r^	9.16 ± 0.26 ^c r^
Ruivo	Ctr	53.79 ± 0.70 ^b s^	5.22 ± 0.14 ^a r^	14.68 ± 0.48 ^d r^	83.14 ± 0.25 ^cd r^	2.36 ± 0.04 ^a r^	11.05 ± 0.11 ^ab r^
	Heat	56.34 ± 0.56 ^bc r^	5.02 ± 0.26 ^a r^	15.41 ± 0.29 ^cd r^	83.47 ± 0.50 ^bc r^	2.20 ± 0.16 ^a r^	11.18 ± 0.49 ^a r^
T94	Ctr	55.60 ± 0.65 ^a r^	5.56 ± 0.12 ^a r^	15.72 ± 0.27 ^cd r^	85.26 ± 0.48 ^bc r^	1.92 ± 0.14 ^a r^	10.13 ± 0.19 ^b s^
	Heat	55.68 ± 0.47 ^cd r^	5.59 ± 0.12 ^a r^	15.46 ± 0.08 ^cd r^	84.82 ± 0.22 ^bc r^	2.43 ± 0.07 ^a r^	11.63 ± 0.06 ^a r^

**Table 6:** Principal component analysis of different traits in wheat.

Principal Component	PC1	PC2	PC3
Proportion of variance	0.2694	0.2109	0.1508
Cumulative proportion	0.2694	0.4803	0.6311
Standard deviation	2.697	2.3861	2.0181

## Data Availability

Data sharing is containing in this article.
